# Absence of Ataxin-3 Leads to Enhanced Stress Response in *C. elegans*

**DOI:** 10.1371/journal.pone.0018512

**Published:** 2011-04-19

**Authors:** Ana João Rodrigues, Andreia Neves-Carvalho, Andreia Teixeira-Castro, Anne Rokka, Garry Corthals, Elsa Logarinho, Patrícia Maciel

**Affiliations:** 1 Life and Health Sciences Research Institute (ICVS), School of Health Sciences, University of Minho, Braga, Portugal; 2 Turku Centre for Biotechnology, University of Turku and Abo Akademi University, Turku, Finland; 3 Instituto de Biologia Molecular e Celular (IBMC), Porto, Portugal; University Medical Center Groningen, The Netherlands

## Abstract

Ataxin-3, the protein involved in Machado-Joseph disease, is able to bind ubiquitylated substrates and act as a deubiquitylating enzyme in vitro, and it has been involved in the modulation of protein degradation by the ubiquitin-proteasome pathway. *C. elegans* and mouse ataxin-3 knockout models are viable and without any obvious phenotype in a basal condition however their phenotype in stress situations has never been described.

Considering the role of ataxin-3 in the protein degradation pathway, we analyzed the effects of heat shock, a known protein homeostasis stressor, in *C. elegans* ataxin-3 (ATX-3) knockout animals. We found that ATX-3 mutants have an exacerbated stress response and survive significantly better than wild type animals when subjected to a noxious heat shock stimulus. This increased thermotolerance of mutants was further enhanced by pre-exposure to a mild heat shock. At a molecular level, ATX-3 mutants have a distinct transcriptomic and proteomic profile with several molecular chaperones abnormally up-regulated during heat shock and recovery, consistent with the observed resistance phenotype.

The improved thermotolerancein ATX-3 mutants is independent of heat shock factor 1, the maestro of the heat shock response, but fully dependent on DAF-16, a critical stress responsive transcription factor involved in longevity and stress resistance. We also show that the increased thermotolerance of ATX-3 mutants is mainly due to HSP-16.2, C12C8.1 and F44E5.5 given that the knockdown of these heat shock proteins using RNA interference causes the phenotype to revert.

This report suggests that the absence of ATX-3 activates the DAF-16 pathway leading to an overexpression of molecular chaperones, which yields knockout animals with an improved capacity for dealing with deleterious stimuli.

## Introduction

Environmental stress often causes proteotoxic damage, which triggers the stress-response machinery in order to maintain cellular homeostasis. Cells have two main lines of defense against misfolded/aberrant proteins: molecular chaperones and the ubiquitin-proteasome pathway (UPP) [1], [2]. Molecular chaperones are responsible for assisting in folding and conformation repair, acting on misfolded proteins to fold them into their native state. Aside from this stress “management” role, some chaperones are expressed constitutively under non-stressful conditions, acting as “protein vigilantes”, monitoring protein quality [3]. The most well-studied chaperones are the Heat Shock Proteins (HSPs), which as the name suggests, respond to heat shock. However, they alsorespond to other types of stressors. The heat shock response is mainly regulated at the level of transcription by Heat Shock transcription Factor 1 (HSF-1) both in mammals and *C. elegans* [4], [5]. Besides being the heat stress response maestro, HSF-1 also influences aging in *C. elegans*, being required for DAF-2–insulin/IGF-1 receptor mutations to extend lifespan [5], [6].

DAF-2 is a transmembrane receptor that acts to negatively regulate the forkhead transcription factor DAF-16 through phosphorylation events [7]. DAF-2 mutants show a lifespan extension that depends on the presence of DAF-16 and increased stress resistance [8], [9]. Interestingly, HSF-1 and DAF-16 act together to activate the expression of specific genes, suggesting cross-talk between the two pathways. The subset of common targets includes genes encoding small heat-shock proteins (sHSPs), which are able to promote longevity and increased stress resistance [5], [6].

When the chaperone machinery fails, the damaged proteins need to be eliminated and are sent for proteasomal degradation. This tight quality control regulation ensures a constant cellular milieu and prevents accumulation of misfolded proteins. However, in aging and certain neurodegenerative conditions such as the polyglutamine (polyQ) diseases, the pathogenic proteins tend to misfold and aggregate, overwhelming the chaperone and UPP machinery, which eventually contributes to neuronal death [10], [11].

One of the putative players in the protein quality control pathway is the polyQ protein ataxin-3, involved in Machado-Joseph disease [12]. Ataxin-3 binds ubiquitin, ubiquitin-like molecule NEDD8, and ubiquitylated proteins. Ataxin-3 is able to act as a deubiquitylating (DUB) enzyme *in vitro* [13], [14], [15], cleaving chains of four or more ubiquitins, which is the minimal signal for proteasomal degradation. Ataxin-3 also associates with HHR23, the UPP-escort protein VCP/p97, UBXN-5 protein and with the proteasome, suggesting a role in the modulation of protein degradation [14], [16], [17], [18]. Through its DUB activity, ataxin-3 can process substrates and either facilitate their proteasomal degradation or rescue proteins from irreversible degradation through the removal of the ubiquitin signal.

Although human, mouse and *C. elegans* ataxin-3 are highly conserved and ubiquitously expressed, the worm and mouse knockout animals do not display any major phenotype [19], [20]. The *C. elegans atx-3* deletion strains are apparently normal, with similar lifespan and brood size when compared to controls in basal conditions [19]. The mouse knockout strain only displays a mild increase in ubiquitylation levels in brain and testis [20]. However, all these studies were performed under basal conditions, and, to our knowledge, nothing is known regarding the behavior of these knockout strains in UPP-demanding situations. Considering the role of ataxin-3 in protein quality control, we decided to analyze the effects of its absence in protein homeostasis stress using *C. elegans* ataxin-3 (ATX-3) knockout strains. Surprisingly, the ATX-3 knockout animals displayed a significantly increased resistance to stress. This improved thermotolerance was due to a higher level of several molecular chaperones, as confirmed by transcriptomic and proteomic analysis, and was fully dependent on the transcription factor DAF-16, but less so on HSF-1. We found that HSP-16.2 was necessary for the increased thermoresistance phenotype of ATX-3 knockout animals, while HSP-16.1 and -16.48 were not.

## Results

### *C. elegans* ATX-3 knockouts have increased resistance to heat stress

Since the *C. elegans* knockoutswere created by random mutagenesis and might bear additional mutation(s), we used two different deletion alleles of ATX-3; both were backcrossed five times to wild-type animals (N2 strain), to exclude other mutations relevant for the phenotype, as previously described [19].

**Figure 1:**
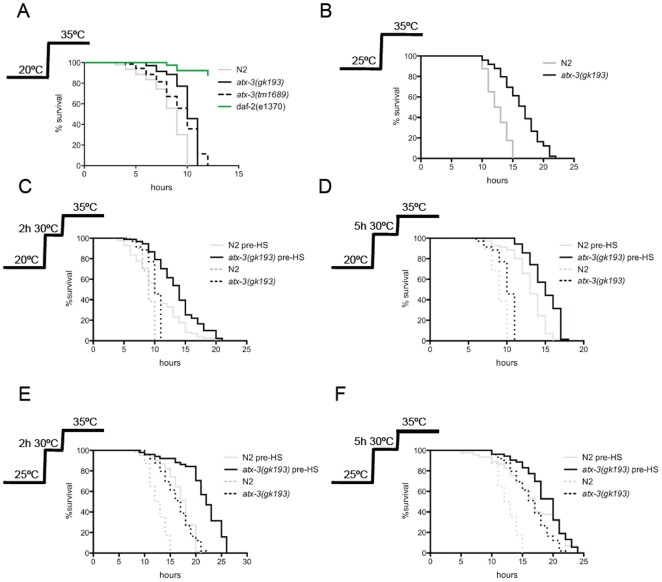
*Atx-3* knockout animals are more resistant to heat stress than wild type. (**A**) Survival curves at 35°C of wild type (N2) and *atx-3* mutants (*gk193*, *tm1689*) previously grown at 20°C (p<0,0001). Median survival of N2 and *atx-3* mutants is 9 h and 10 h respectively. (**B**) Survival curves at 35°C of N2 and *atx-3* mutantspreviously grown at 25°C (p<0,0001). Median survival of N2 and *atx-3* mutants is 12.5 h and 17 h respectively (p<0,0001). Pre-exposure to a non-lethal heat shock of 2 h (**C**) or 5 h (**D**) at 33°C further enhances the thermoresistance phenotype of *atx-3* mutants (p<0,0001). Median survivals are: N2: 9 h, *atx-3*: 10 h, N2 pre-HS_2h_: 10 h, *atx-3* pre-HS_2h_: 14 h, N2 pre-HS_5h_: 13 h, *atx-3* pre-HS_5h_: 15 h. The same trend is observed with animals previously grown at 25°C (p<0,0001) both with the 2 h (**E**) and 5 h (**F**) pre-HS. Median survivals are: N2: 12.5 h, *atx-3*: 17 h, N2 pre-HS_2h_: 18 h, *atx-3* pre-HS_2h_: 22 h, N2 pre-HS_5h_: 17 h, *atx-3* pre-HS_5h_: 20 h. One representative experiment is shown (at least three independent replicates were performed).

Interestingly, as depicted in Figure 1A, ATX-3 knockout animals grown at 20°C exhibited a significant higher rate of survival compared to wild-type animals when exposed to a lethal heat shock at 35°C. Wild-type animals displayed a median life span of 9 hours, while both mutant strains had a median lifespan of 10 hours (p<0,0001), a 10% increase in survival. As both mutants behaved similarly with regards to their thermotolerance, we decided to use only the*gk193* allele for further studies. As expected, *daf-2* mutants, who are known to be long-lived and stress-resistant [8], [9], [21], [22], lived significantly longer than both strains with more than 80% of animals living when no wild type or atx-3 animals remained.

In addition to analyzing the animals under baseline conditions (20°C), we also grew the *atx-3* mutants at a stress-threshold situation, at 25°C, and analyzed their survival when subjected to a 35°C heat shock. Regardless of the genotype, all strains displayed enhanced survival at 35°C compared to the same strains grown at 20°C. Similarly to the basal condition, *atx-3* null animals survived significantly better than wild type animals, with a median survival time of 17 hours versus 13 hours respectively, representing an increase of 30% in *atx-3* knockout animals (p<0,0001) (Figure 1B). Table with lifespan results and p values in Table S1.

### Increased resistance to stress is further enhanced by hormesis

To investigate whether the stress machinery could be activated more rapidly/efficiently in *atx-3* mutants, we performed an additional experiment, in which animals were pre-exposed to a non-lethal heat shock at 30°C and then transferred to the lethal temperature (35°C). This pre-exposure at 30°C is known to stimulate protective cellular mechanisms and improve the organism's ability to cope with that and other types of stress in a process known as hormesis, which occurs in several animal species including worms [23].

We pre-exposed the animals to the sub-lethal heat shock for 2 or 5 hours and then transferred these animals to the lethal temperature and measured their survival. After a pre-heat shock for 2 hours at 30°C, the survival curves of both wild type and *atx-3* mutants shifted to the right, representing an increased survival. However, the *atx-3*-null animals were significantly more resistant than wild type, with a median lifespan of 14 hours compared to 10 hours in wild type, corresponding to an increase of 40% in survival time in mutants (p<0,0001) (Figure 1C). When we pre-exposed the animals to a 5-hour pre-heat shock treatment, the median lifespans were 15 hours and 13 hours for *atx-3* and wild type animals, respectively. These values corresponded to a 13% increase in survival time among mutants (p<0,0001) (Figure 1D).

When animals grown at 25°C were subjected to the same protocol, with the 2-hour pre-heat shock, *atx-3* mutants' median survival was 22 hours compared to 18 hours of N2 animals (22% increase) (p<0,0001) (Figure 1E). In the case of the 5-hour pre-treatment, the knockout animals' median survival was 20 hours versus 17 hours for N2 animals (18% increase) (Figure 1F).

### *atx-3* knockouts show distinct changes in chaperone mRNA levels

The next step was to try to understand the mechanism of this increased resistance at a molecular level. To do so, we analyzed the gene expression of several chaperones in the *atx-3* mutant strain, including three small heat shock proteins (sHSP) (HSP-16.49, HSP-16.1, HSP-16.2) and four members of the HSP70 family (F44E5.5, HSP-4, HSP-1, C12C8.1), using real-time PCR.

**Figure 2:**
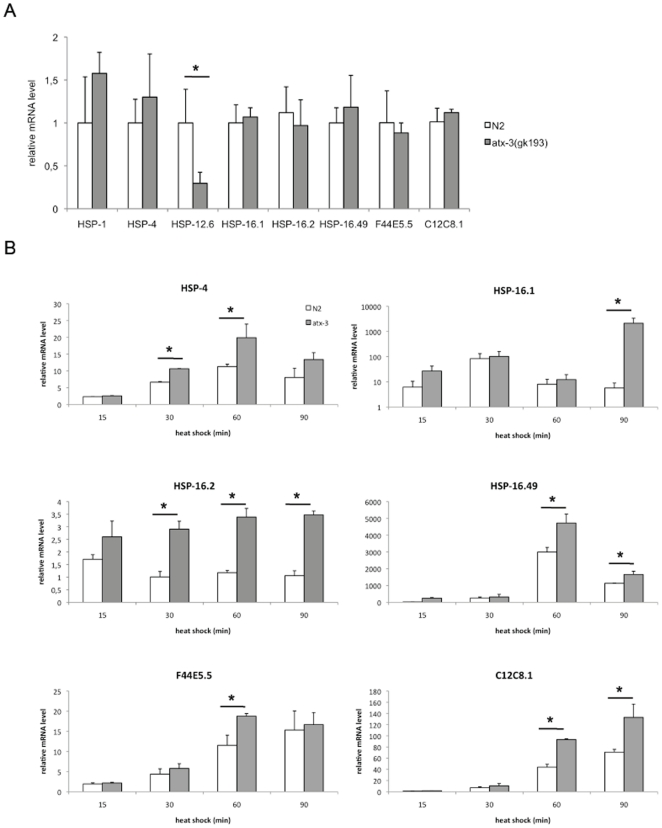
*Atx-3* knockout animals display increased up-regulation of molecular chaperones upon heat stress at mRNA level. (**A**) At 20°C, atx-3 mutants do not show any significant difference in the levels of several molecular chaperones, although HSP-12.6 shows a trend to be down-regulated. (**B**) Atx-3 mutants have mRNA up-regulation of several chaperones during the course of heat shock (HS) as measured by Real-Time PCR in young adult animals. HSP-4 was up-regulated from 30 until 60 minutes after the beginning of the HS and returned to wild type levels at 90 minutes. HSP-16.1 was only significantly increased 90 minutes after the stimulus while HSP-16.49, F44E5.5 and C12C8.1 were up-regulated at 60 minutes. Error bars correspond to standard error. * p<0.05.

*Atx-3* mutants displayed no pre-activation of the chaperone machinery at 20°C, as determined by analyzing mRNA expression (Figure 2A).

When we analyzed the chaperone expression levels during the course of a standard *non-lethal* 33°C heat shock, we found that most of the chaperones tested were significantly up-regulated in *atx-3* mutants 60 minutes after the beginning of the heat shock. The expression of HSP-16.2 and -4 was augmented in mutants 30 minutes after heat shock was initiated, while HSP-16.49, F44E5.5 and C12C8.1 up-regulation was obvious at 60 minutes. HSP-16.1 was significantly up-regulated in *atx-3* mutants only 90 minutes after the stimulus (Figure 2B).

HSP-1, -12 and DAF-21 (HSP-90 homologue) levels did not differ between wild type and *atx-3* mutants during the time-course of the heat shock (data not shown).

### Proteomic profile of *atx-3* mutants after heat shock

**Figure 3:**
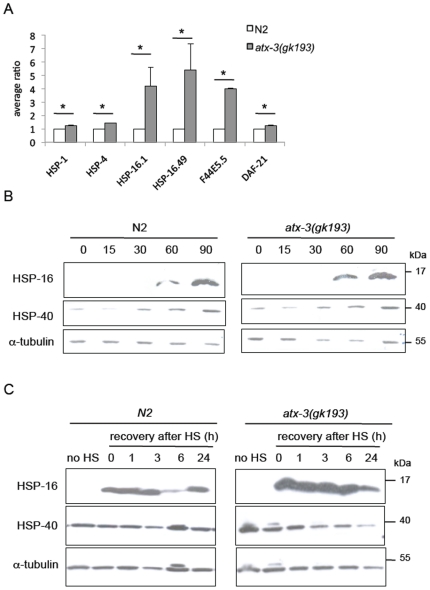
Absence of *atx-3* leads to up-regulation of several molecular chaperones during heat shock and in the recovery at a protein level. (**A**) Summary of the proteomic results regarding the expression levels of several chaperones after heat shock of 2 h and recovery of 30 minutes at 20°C. Shown is the average of the relative expression of chaperones normalizing wild type levels to 1. Error bars correspond to standard deviation. (**B**) Western blot analysis of constitutive (HSP-4) and inducible chaperones (HSP-16 family) in wild type and *atx-3* animals during the timecourse of a heat shock. HSP-40 is expressed at all timepoints while the HPS-16 family of proteins is only detected at 60 minutes after the beginning of the heat shock. Mutants have higher levels of HSP-16 from 60–90 minutes after the beginning of the stimulus. (**C**) Atx-3 mutants have the HSP-16 family up-regulated during the timecourse of the recovery. Only 24 h after heat shock, the HSP-16 levels become similar to wild type. * p<0.05.

Real-time PCR results suggested that the *atx-3* strain exhibited an enhanced activation of the chaperone machinery, at least at the mRNA level, during the heat shock. Our next step was to analyze the proteomic profile of *atx-3* after a standard non-lethal heat shock in *C. elegans* (2 h at 33°C). Detecting and quantifying whole proteins from a complex protein extract in a comprehensive manner remains a challenge in the fields of proteomics, nevertheless, using the iTRAQ technique, which allows simultaneous quantification of 2–8 samples by using different isotopes (Applied Biosystems), we were able to obtain acceptable (albeit incomplete) results. In the baseline condition (20°C), 35 proteins were altered in the *atx-3* knockout animals when compared to wild type (Table S2). These proteins belong to several heterogeneous classes such as ribosomal proteins (rpl-5, -33, -20), vitelogenins (vit-1, -4, -5) and histones (his-12, -14, -71). After heat shock, 148 proteins were significantly altered; with a predominance of ribosomal proteins, molecular chaperones, enzymes and histones. There was partial overlap between these two conditions in terms of protein alterations in *atx-3* mutants (rpl-5, rpl-20, his-71, hi-12, his-14, vit-1, vit-5, vit-4, sodh-1). Interestingly, as depicted in Figure 3A, both *atx-3* knockout strains revealed a significant increase in the levels of the molecular chaperones HSP-16.49, HSP-16.1 and F44E5.5. Although the levels of HSP-1, HSP-4 and DAF-21 (HSP90 homologue) were only slightly up-regulated, these differences were statistically significant. We observed the same pattern of changes in both *atx-3* mutant strains (see Table S2 for complete proteomic results).

The most consistent difference was in the expression of HSP-16 family members - HSP-16.1 and HSP-16.49, which were clearly up-regulated in *atx-3* strains. We were unable to quantify the levels of HSP-16.2 protein. The SIP-1 protein (sHSP) was altered in the knockout strains but displayed a divergent profile in the biological replicates we analyzed (data not shown). Other chaperones such as HSP-3, -6, -12.2 and -60 were present at similar levels in wild type and *atx-3* mutant animals while HSP-12.6 levels were diminished (Table S2).

We aimed to confirm some of these findings by western blot. Aside from analyzing the time-course of the heat shock, as previously performed using real-time PCR, we also analyzed the chaperone profile of the recovery after heat shock. We were able to confirm HSP-16 overexpression using anti-HSP16 antibody (kindly provided by Dr. Christopher Link). Since this antibody recognizes several members of the HSP-16 family, the overexpression detected (Figure 3B) was less pronounced than in the proteomic analysis, probably due to the masking effect of other proteins of this family, which were unaltered in the *atx-3* animals. We found that HSP-16 proteins were first detected 60 minutes after the beginning of heat shock. HSP-16 levels were elevated in *atx-3* animals when compared to controls at 60–90 minutes of heat shock. We also analyzed the expression levels of HSP-40, a constitutive chaperone, and we found no differences between *atx-3* and N2 animals.

Several time-points of recovery after heat shock were also analyzed: 1 h, 3 h, 6 h and 24 h. The HSP-16 proteins remained detectable at all time-points and were found to be consistently up-regulated in *atx-3* nulls (Figure 3C), although at 24 h, levels were comparable to those observed inwild type animals.

We tested various commercial antibodies against other chaperones (hsp70, hsp105) with no success, since they did not recognize the worm proteins (data not shown).

### *atx-3* mutant animals have a lower temperature threshold for the induction of chaperones

**Figure 4:**
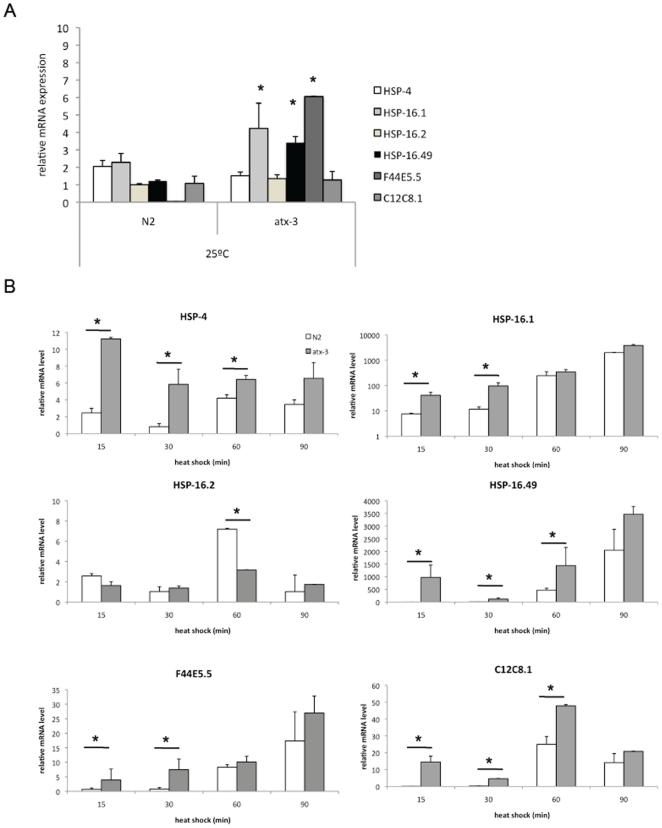
*Atx-3* mutants have a lower threshold for induction of heat sock response. (**A**) When grown at 25°C, atx-3 animals present HSP-16.1, -16.49 and F44E5.5 up-regulated when compared to control animals (p<0.05). (**B**) Atx-3 mutants have mRNA up-regulation of several chaperones during the course of heat shock (HS) as measured by Real-Time PCR in young adult animals previously grown at 25°C. HSP-4 was up-regulated in mutants from 15 until 60 minutes after the beginning of the HS and returned to wild type levels at 90 minutes. HSP-16.1 was only significantly increased during the first 30 minutes; HSP-16.49 and C12C8.1 expression levels were increased from 15–60 minutes while F44E5.5 was up-regulated until 30 minutes after the beginning of HS. Error bars correspond to standard error. * p<0.05.

Our next step was to assess how these animals would behave if grown at a stress-threshold temperature, at 25°C. At 25°C, *atx-3* mutants exhibited significantly increased levels of mRNA for the inducible HSP-16.1 and -16.49 chaperones, as well as F44E5.5 in comparison to N2 animals (Figure 4A). No changes were observed in HSP-16.2 and C12C8.1 expression levels. This “pre-activation” of the chaperone machinery, however, is probably not translated at the protein level, since we were unable to detect HSP-16 proteins by western blot at this temperature (data not shown).

**Figure 5:**
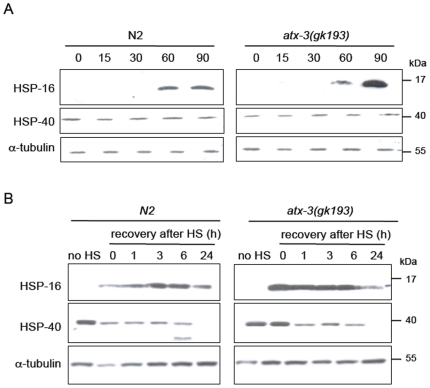
*Atx-3* knockout animals display significant changes in the levels of molecular chaperones when grown at 25°C. (**A**) Western blot analysis of constitutive (HSP-4) and inducible chaperones (HSP-16 family) in wild type and *atx-3* animals during the timecourse of a heat shock. HSP-40 is expressed at all timepoints while HPS-16 is only detected 60 minutes after the beginning of the heat shock. Mutants have higher levels of HSP-16 from 60–90 minutes after the beginning of the stimulus. (**C**) *Atx-3* mutants have the HSP-16 family up-regulated during the timecourse of the recovery. Only at 24 h, the HSP-16 levels return back to wild type levels.

We also analyzed the HSP mRNA and protein expression levels during and after heat shock in animals grown at 25°C. As depicted in Figure 4B, mRNA levels of HSP-4, -16.1, -16.49, F44E5.5 and C12C8.1 were up-regulated in *atx-3* animals at 15 minutes after the beginning of heat shock until at least 30 minutes after the stimulus. At the protein level, *atx-3* animals expressed higher levels of HSP-16 than controls 90 minutes after the beginning of heat shock, and these proteins remained up-regulated at all recovery time-points except at 24 h, when the HSP-16 levels were close to those of wild-type (Figure 5A, B). Surprisingly, the levels of so-called “constitutive” HSP-40 varied in some experiments; in fact, it was often undetectable 24 h after heat shock (Figure 5B), which suggests that it may also be regulated by stress. Interestingly, HSP-16.2 was not up-regulated in *atx-3* mutants under these conditions and was even down-regulated 60 minutes after the beginning of heat shock (Figure 4A, B).

### Increased resistance to stress but no extension in lifespan in *atx-3* mutants

**Figure 6:**
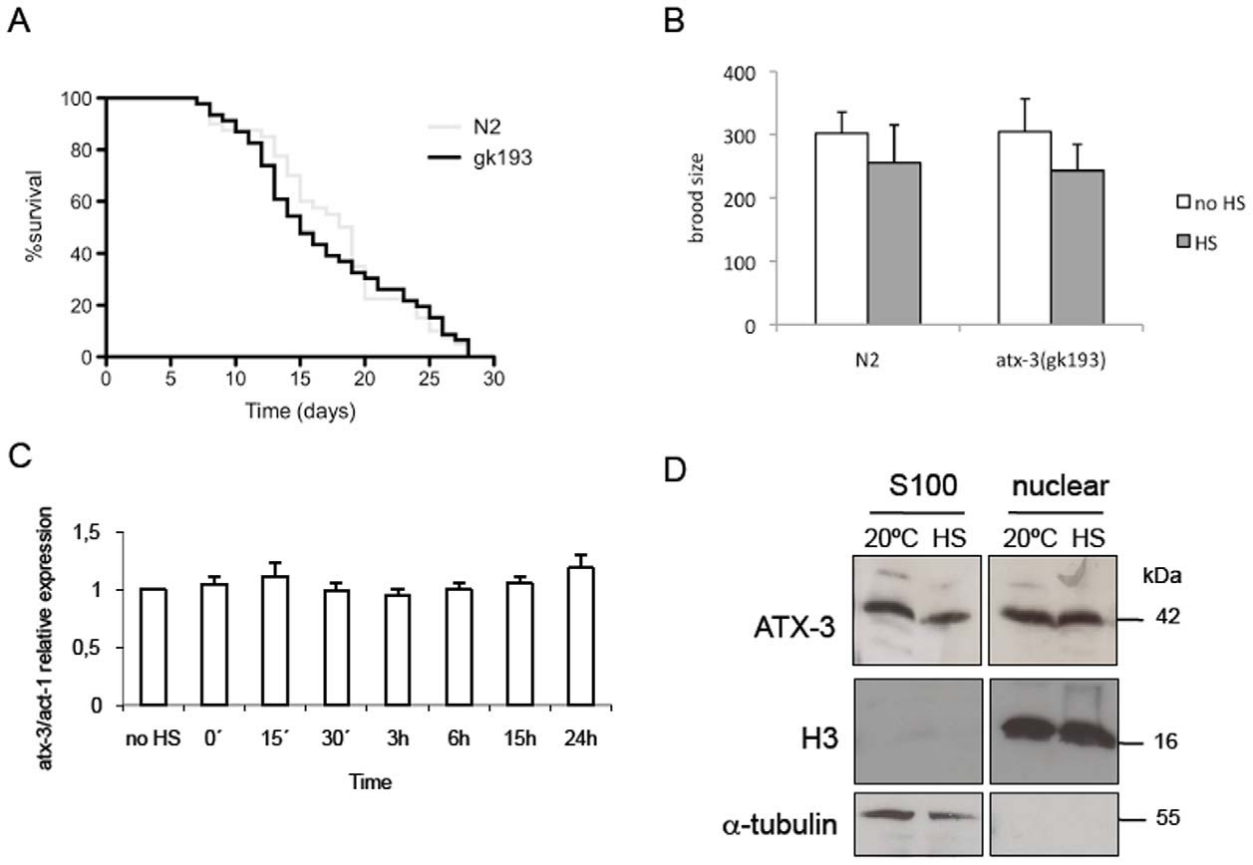
*Atx-3* expression does not change with stress. (**A**) Exposure to heat shock early in life does not extend *C. elegan*s*atx-3* mutants lifespan nor does it change the number of progeny when compared to wild type (**B**). (**C**) *Atx-3* mRNA levels remain similar after a heat shock in *C. elegans*. No HS: no heat shock, 15′: 15 minutes of recovery at 20°C after the 2 h HS at 33°C, 30′: 30 minutes of recovery, 3 h–24 h: 3 hours-24 hours of recovery. (**D**) No differences in ATX-3 protein levels were detected in the cytoplasm and in the nucleus, neither in a basal condition nor after heat stress. H3: histone H3; S100: cytoplasmic fraction.

Although stress-resistant animals are usually long-lived [24] and a raise in chaperone levels is often correlated with increased lifespan [25], [26], we have previously shown that *atx-3* mutants have normal lifespan [19]. However, we had not analyzed their lifespan after a heat insult; hence, we exposed the mutant animals to a 33°C heat shock for two hours and then transferred them to 20°C, checking their survival daily. *Atx-3* animals lived for the same amount of time as the wild type after the exposure to heat shock (Figure 6A). The brood size of these animals after heat shock was also similar to that of wild type (Figure 6B).

Since *atx-3* knockout animals displayed a clear resistance to stress, we decided to assess the levels of *atx-3*mRNA after exposure to heat shock, in order to see if *atx-3* behaved as a negative stress-responsive gene, being repressed upon exposure to stress. As can be seen in Figure 6C, the number of transcripts of *atx-3* was not altered significantly after the insult. Since it has been shown that human ataxin-3 is translocated to the nucleus in cell lines exposed to heat shock, we also assessed the subcellular distribution of ATX-3 protein in a baseline condition and after heat shock in *C. elegans*. In contrast to the above-mentioned observations, no differences were found in nuclear ATX-3 levels in both conditions (Figure 6D).

### *atx-3* modulates stress resistance in a *daf-16*-dependent manner

**Figure 7:**
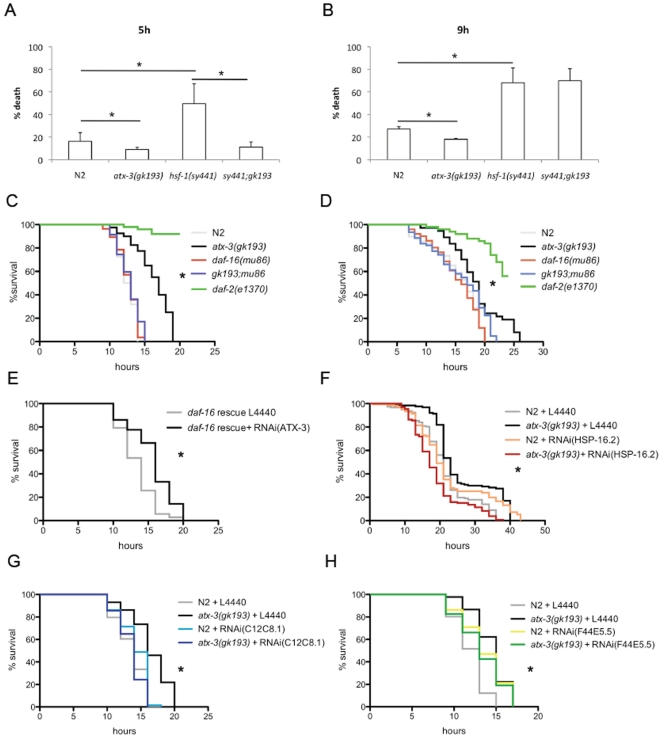
*Atx-3* thermoresistance is dependent on DAF-16 transcription factor and independent on HSF-1. Wild type animals die more than *atx-3* mutants after a 5 h (**A**) or 9 h (**B**) exposure to 35°C heat shock. *Hsf-1* animals have enhanced sensitivity to this temperature at both timepoints, as expected. Double *atx-3; hsf-1* mutants still display enhanced thermoresistance at 5 h timepoint but behave as single *hsf-1* mutants in the 9 h heat shock condition. *Atx-3* mutants are more resistant to the exposure to a lethal heat shock at 35°C than wild type animals, but *daf-16*;* atx-3* double mutants are not, both when grown at 20°C (**C**) or 25°C (**D**). (**E**) Restoring DAF-16 levels in a *daf-16*;* atx-3* (*daf-16* deletion and *atx-3* knockdown; *daf-16* rescue strain) background leads to the appearance of the thermotolerance phenotype. Increased thermotolerance of *atx-3* mutants is still observed when grown on bacteria expressing RNAi empty vector (L4440) (**F**) and is dependent on *hsp-16.2* (**G**), C12C8.1 (**H**) and F44E5.5 (**I**). Median survivals in animals grown in (C) are: N2: 12.5 h, *atx-3*: 17 h, *daf-16*: 13 h, *daf-16*;* atx-3*: 13 h, *daf-2*: not determined. Median survivals in (D) are: N2: 17 h, *atx-3*: 19 h, *daf-16*: 16 h, *daf-16*;* atx-3*: 17 h, *daf-2*: not determined. Median survivals in (E) are: *daf-16* rescue in L4440: 14 h, *daf-16* rescue in RNAi(ATX-3): 16 h. Median survivals in (F) are: N2 L4440: 21 h, *atx-3* L4440: 23 h. N2 in RNAi(HSP-16.2): 19 h, *atx-3* in RNAi(HSP-16.2): 17 h. Median survivals in (G) are: N2 L4440: 14 h, *atx-3* L4440: 16 h. N2 in RNAi(C12C8.1): 14 h, *atx-3* in RNAi(C12C8.1): 14 h. Median survivals in (H) are: N2 L4440: 13 h, *atx-3* L4440: 15 h. N2 in RNAi(F44E5.5): 13 h, *atx-3* in RNAi(F44E5.5): 13 h. *p<0.05.

In order to further understand the mechanism of improved stress resistance in *atx-3* knockout animals, we decided to analyze whether *atx-3* could genetically interact with the transcription factors HSF-1 and DAF-16, which are important for lifespan and stress resistance and have been shown to co-regulate sHSP expression together [5]. To do so, we crossed *atx-3* animals with worms bearingan *hsf-1* point mutation allele (*hsf-1(sy441)*), which results in a truncated version of HSF-1, unable to activate transcription [27].To assess the thermotolerance profile of *hsf-1;atx-3* animals, we exposed them to varying durations of 35°C heat shock and 12 h later analyzed their survival, a protocol adapted from thatdescribed previously [28]. The thermoresistance phenotype of *atx-3*-null animals was independent of the HSF-1 transcriptionfactor, since*hsf-1; atx-3* double-mutants behaved as *atx-3* single-mutants when exposed to a 5 h heat shock (Figure 7A, B). However, after a 9 h heat shock, the double mutant animals no longer displayed this increased thermotolerance, suggesting that the phenotype is partially dependent on HSF-1 (Figure 7B). We were unable to perform this assay with animals grown at 25°C, given that the *hsf-1*-null animals are extremely sensitive to temperatures above 20°C. The thermoresistance phenotype of *atx-3* mutants, however, was completely dependent on DAF-16, as the *daf-16; atx-3* double-knockout animals behaved as single *daf-16* mutants in the thermotolerance assay (p = 0,18) (Figure 7C). This *daf-16* phenotype-dependence was observed with animals grown at 25°C as well (Figure 7D). To further confirm the dependence of DAF-16 for the observed resistance phenotype, we used a rescue strain (here named *daf-16* rescue) that is homozygous for the mgDf47 mutation in the *daf-16* gene and contains an integrated plasmid with DAF-16B::GFP under the control of the endogenous promoter [7]. Knockdown of *atx-3* by RNAi in this rescue background restored the enhanced thermotolerancephenotype (Figure 7E). The results presented so far suggest an activation of DAF-16 with concomitant overexpression of the molecular chaperones. In support of this hypothesis was the fact that *sod-3* and *mtl-1* (other DAF-16 transcriptional targets) were also up-regulated in *atx-3* mutants (Figure S1).

To identify the molecular chaperones important for the observed phenotype, we performed RNA interference (RNAi) against several HSPs. As depicted in Figure 7, *atx-3* mutants were still more resistant than wild type when grown in bacteria transformed with L4440 (RNAi empty vector) (Figure 7F). Gene knockdown of *hsp-16.1* and *hsp-16.49* did not revert the increased thermotolerance phenotype of *atx-3* mutants (data not shown), while knockdown of *hsp-16.2* reverted this phenotype (Figure 7F). In addition, RNAi of the hsp70 family members C12C8.1 and F44E5.5 also reverted thethermotolerance phenotype (Figure 7G, H; complete results in Table S3).

## Discussion

Ataxin-3 is a DUB enzyme involved in the ubiquitin-proteasome pathway, where it seems to act as a “processing” protein, cleaving ubiquitin from substrates and modulating their proteasomal degradation [14], [16], [17], [18]. Ataxin-3 is ubiquitously expressed and is present in several species, ranging from plants to humans, as well as in almost every type of cells. Therefore it is quite surprising that the ataxin-3 knockout mice only display a mild increase in ubiquitylation levels and that a *C. elegans* model of ataxin-3 deficiency shows no obvious phenotype and no differences in lifespan, brood size or general ubiquitylation levels [19], [20]. This could indicate adaptive/compensatory mechanisms or very limited substrate specificity. Although the ataxin-3 knockout models are apparently normal in baseline conditions, little is known about the behavior of these mutants under stress. Recently, we have shown that *C. elegans atx-3* mutants display a temperature-dependent motor phenotype [29].

Since this protein shares the sequence, structure and biochemical activity of its human counterpart [19], we studied the effects of ATX-3 absence in *C. elegans* under stressconditions. We observed that ATX-3 knockout animals are significantly more resistant to heat stress than wild type animals. Our first hypothesis was that the *atx-3* gene might be a stress-responsive gene/protein, potentially with a role in termination of the stress response mediated by degradation of specific substrate proteins. However, this hypothesis is contradicted by the fact that heat shock did not change *atx-3* expression levels and ATX-3 subcellular distribution in *C. elegans*.

Interestingly, while this manuscript was being prepared, it was shown that in fibroblasts, ataxin-3 responds to stress by moving to the nucleus, and that knockout fibroblasts were more sensitive to heat stress than controls [30], which is in contrast with our findings. Although it can be argued that the absence of ataxin-3 in mammals and worms can lead to dissimilar consequences, this seems unlikely as the two proteins have the same biochemical function, localization and expression pattern [19]. More likely to explain the differences is the fact that the effects of heat shock in a cell line are certainly different from those in a whole organism such as *C. elegans*. In isolated cells, the heatshock response is initiated by the presence of misfolded proteins (cell-autonomous), while in *C. elegans*, the heatshock response of somatic cells depends on the thermosensoryneuron AFD which regulates temperature-related behavior [31].

Underlying thethermoresistance phenotype of *atx-3* mutants was the significant up-regulation of several molecular chaperones, namely HSP-1, HSP-16.1, HSP-16.2, HSP-16.49, HSP-4, F44E5.5, C12C8.1 and DAF-21. This up-regulation was observed after heat shock in animals grown at 20°C and, for most of the molecular chaperones listed above when animals were grown at 25°C as well. Increased levels of HSPs have a protective effect and enhance the stress response in *C. elegans*. For example, elevated levels of HSP-16 increase stress resistance [26] and can predict both thermotolerance and survival of individual worms [32]. Overexpressing other HSPs such as HSP-70 also induces increased resistance to stress in worms [33].The increase in HSPs in *atx-3* mutants seems to occur only after stress since proteomic analysis did not reveal any difference under baseline conditions. Interestingly, the heat-resistance phenotype was further enhanced by a non-lethal pre-heat shock, which may indicate that the stress machinery is somehow more efficiently activated in knockout animals. Consistent with this hypothesis is the fact that the differences between mutants and controls are more evident with the 2 hour than with the 5 hour pre-treatment, suggesting that a 2 hours heat shock activates the stress-machinery (i.e. chaperones) system in both strains, although in mutants to a greater extent, whereas a 5 hour pre-heat shock may “saturate” the stress machinery, which reaches a plateau in both strains, masking the improved response of the mutants. The analysis of chaperones, at the mRNA level at 25°C, suggests that the *atx-3* null animals have a lower temperature threshold to induce the transcription of stress-responsive genes as the sHSPs, supporting this hypothesis.

Although there is a strong correlation between increased stress resistance, high chaperone levels and lifespan extension in *C. elegans* [5], [25], [26], [34], we found no differences in the lifespan of *atx-3* mutants, even when the chaperone machinery was strongly activated early in life. These results are consistent with the fact that the chaperones were not altered in basal conditions in the knockout animals, which is typical of long-lived strains [5]. Other mutant strains like *daf-4* and *daf-7*, havealso been described to be thermotolerant but not long-lived [22].

Another remarkable finding in the proteomic analysis is that, of the detected chaperones, those that were not altered in the mutants are either constitutively expressed or only induced upon very specific types of stress such as mitochondrial stress for example (HSP-3, -6, -60) [35], [36]. The up-regulated proteins in *atx-3* knockout animals were mainly those induced by *general* stress- HSP-16.1, -16.49, -1 (weak), -4 [35], [37], [38], which can explain the absence of differences in basal conditions. It is also noteworthy to mention that an ER-chaperone, HSP-4, was up-regulated in *atx-3* knockout animals, since it has been proposed that ATX-3, along with its interactor VCP/p97 protein, is involved in ER-associated protein degradation [29]. *C. elegans* knockouts for calreticulin and calnexin, important ER-resident chaperones, show an increase in the ER-chaperone HSP-4 and a decrease in the SODH-1 protein [39], as we observe for the *atx-3* knockout animals, suggesting a similar molecular compensation mechanism may be occurring in both mutants and reinforcing the hypothesis of an involvement of ATX-3 in ERAD, although more studies have to be performed to validate this theory.

In *C. elegans*, HSF-1 and DAF-16 together activate the expression of specific stress genes, of which the sHSPs,suggesting a cross-talk between the two pathways [5].But although there is an evident connection between these two factors, neither DAF-16 nor HSF-1 is completely required for each other's activity. For example, loss of HSF-1 does not prevent DAF-16 accumulation in the nuclei of *daf-2* mutants nor the activation of two DAF-16 target genes (*mtl-4* and *sod-3*). Similarly, the activation of HSF-1 targets such as *aip-1*, *unc-33* and F44E5.4 is independent from DAF-16 [5]. Here, we demonstrate that the thermoresistance phenotype of *atx-3* strains is independent on HSF-1, at least to a certain extent. This finding was surprising given the fact that the HSF-1 is a major player in the stress response; however, it has also been shown that DAF-16 *per se* is able to activate molecules of the stress response. Thus, it was interesting to find thatthe *atx-3* mutants' phenotype was fully dependent on DAF-16. Double *daf-16; atx-3* mutants behaved exactly as single *daf-16* mutants in the thermotolerance assays but the rescue strain displays enhanced thermotolerance, suggesting that this transcription factor is required to mount the enhanced stress response in *atx-3* mutants. While this manuscript was being prepared, Hoppe and colleagues showed that *cdc-4; atx-3* double knockouts were long lived (up to 50%) and this seemed to be mediated by the DAF-16 pathway [40]; this relationship between ataxin-3 absence and DAF-16 activation is in accordance with our findings.

When we further dissected the molecular events responsible for this thermoresistance phenotype, we found that the *hsp-16.2* gene, a DAF-16 target, was essential for the increased survival of *atx-3* strain: the *atx-3* knockout animals not only lost their increased thermoresistance but also displayed more sensitivity than wild type animals to the lethal temperature when subjected to *hsp-16.2* RNAi. *Hsp-16.2* encodes a sHSP similar to *sip-20* (*s*tress *i*nduced *p*rotein 20) which is activated in response to heat shock and other stressors. Expression of *hsp-16.2* is a good predictor of stress response and longevity [32] and it has been shown to reduce the aggregation of beta amyloid peptide *in vivo* [41]. Besides, *hsp-16.2* expression is known to be modulated by HSF-1 and by DAF-16 [5], [34]. The finding that *hsp-16.2* was essential for the phenotype was quite surprising since although its expression was higher after heat shock in *atx-3* animals when compared to controls when grown at 20°C, the same was not observed with animals grown at 25°C, at least at the mRNA level. One possibility is that the down-regulation result from negative feedback by accumulated HSP-16.2 at the protein level, a hypothesis we not verified due to the lack of HSP-16.2 specific antibody. For example, Hsp70 is able to function as a repressor of the heat shock response in eukaryotes [42] and in bacteria, chaperones DnaK, DnaJ and GrpE negatively regulate the transcription of heat shock genes [43], [44]. In addition to HSP-16.2, we found that C12C8.1 and F44E5.5, which were significantly up-regulated after heat shock, were also essential for the thermoresistance phenotype of *atx-3* mutants. C12C8.1 and F44E5.5 are hsp70 members, highly activated after heat shock [37] and apparently regulated by HSF-1 [31]. Although it appears that their expression is not modulated by DAF-16, the existence of a parallel transcriptional mechanism cannot be ruled out [31]. Noteworthy, RNAi against other chaperones did not lead to phenotype reversion, enhancing the specificity of the abovementioned chaperones for the observed thermotolerance.

The previously described translocation of ataxin-3 into the nucleus of cell lines following heat shock has been shown to be independent of HSF-1 [30], which suggests that alternative pathways may be related to ataxin-3's potential involvement in the stress response. At least in *atx-3* animals, it seems that chaperone overexpression is being activated mainly by DAF-16, given the requirement of DAF-16 for the phenotype. Nevertheless, the *hsf-1; atx-3* mutants still require HSF-1 in the longer heat shock situation, suggesting that after a certain degree of damage/exposure, both pathways are necessary.

One possible explanation for the molecular and physiological phenotype of *atx-3* nulls is that the absence of ataxin-3 at some timepoint of the development causes cellular (proteotoxic?) stress, which activates the stress machinery, and once they are needed again, chaperones and other effectors will be more effectively and rapidly activated- a process known as hormesis. Another possibility is that ataxin-3 is normally regulating chaperone levels via the DAF-16 pathway, or even modulating their levels through the ubiquitin-proteasome degradation of a specific target upstream DAF-16 or of DAF-16 itself. This last option seems unlikely as we did not find significant differences in DAF-16 protein levels in *atx-3* mutant animals (data not shown), in agreement with very recent findings [40].

In summary, we show that the absence of ataxin-3 leads to an enhanced stress response in *C. elegans*. This phenotype was correlated with a significant increase in chaperones and fully dependent on the transcription factorDAF-16 and on its target HSP-16.2, and on the hsp70-like C12C8.1 chaperone.These findings can be relevant in the disease context, since a partial loss of the normal function of ataxin-3 may occurdue to the expansion, as has been observed for other polyQ disorders. Long-term deregulation of HSPscan be detrimental for cell growth, division and viability [45], [46] and, along with the proteotoxic stress, this may contribute to neuronal demise in the context of MJD.

## Materials and Methods

### *C. elegans* strains culture and crosses

*C. elegans* strains were maintained at 20°C or 25°C in 2% peptone or NGM plates seeded with *E. coli* OP50 (28). Synchronous cultures were obtained by bleaching.

Strain *atx-3(gk193)* was kindly provided by the *C. elegans* Reverse Genetics Core Facility at the University of British Columbia. Strain *atx-3(tm1698)* was obtained from the National Bioresource Project. Strain GR1352 (*daf-16(mgDf47)* I; xrIs87 [daf-16alpha::GFP::DAF-16B+rol-6(su1006)] was kindly provided by Dr. Gary Ruvkun. Other strains used in this work were kindly provided by the *Caenorhabditis* Genetics Center(CGC).

Strain *atx-3(gk193)* was crossed with strain *daf-16(mu86)* and *hsf-1(sy441)* using standard procedures. *Daf-16* genotyping was done using primers mu86+U5311, mu86+L16642 and mu86-L6429 as previously described [47]. An allele specific PCR using primers sy441F1, sy441F2 and sy441R1 was performed for *hsf-1* genotyping. *Atx-3* genotyping was described before [19]. Primer sequences in Table S4.

### *C. elegans* growth conditions

For the heat shock experiments, young adult animals were submitted to the indicated temperature in a temperature-controlled incubator, and after the desired period, animals were collected as described above.

### *C. elegans* life span and brood size assays

For regular life span, animals were kept at 20°C and checked daily for viability. Animals were also subjected to a 2 h 30°C heat shock and then transferred to 20°C and checked daily. For brood size measurements, animals were allowed to put eggs at 20°C with or without a heat shock of 2 h at 33°C. Progeny was allowed to eclode and counted the next day.

### RNAi experiments

Genes of interest cloned in L4440 vector were grown from standard *C. elegans* libraries and verified by sequencing. L4440 empty vector was used as a control. L4440/*mex-3* was used as positive RNAi control (easy to score phenotype). HT115 bacteria transformed with L4440 with gene of interest or empty L4440 vector were grown in standard LB media with ampicillin (50 µg/ml) overnight at 37°C. The next day, bacteria were seeded onto LB+ampicillin plates supplemented with 1 mM IPTG to induce expression. 2–3 days later, synchronized L1 animals were transferred to plates, placed at the correct temperature and checked every day.

### Thermotolerance assays

For regular thermotolerance assays, synchronized young adult animals (72 h post hatching) were grown at 20°C or 25°C and then transferred to 35°C and checked every hour for death animals. In the case of pre-heat shock, animals were initially submitted to 30°C for two or five hours and immediately transferred to 35°C and checked every 1–2 hours. To test HSF-1 phenotype dependence, animals were subjected to a 35°C heat shock for 5–9 hours, transferred to 20°C for recovery and scored for dead animals 12 hours later [31]. More than 50 animals per group were analyzed in each experiment and at least three independent replicates were performed for each assay.

### Western Blotting

Synchronous worm cultures were collected with M9 buffer and rinsed several times. After that, they were pelleted, frozen in liquid nitrogen. An equal amount of Lysis buffer (50 mMTris-HCl pH 7.4, 150 mMNaCl, 1% NP-40, 1 mM PMSF, complete protease inhibitors (Roche)) was added to each pellet. Animals were frozen and thawed several times, briefly sonicated and after centrifugation at 13000 rpm 10 minutes at 4°C, the supernatant was collected. Proteins were quantified using the Bradford method. Forty micrograms of total protein was loaded into SDS-PAGE gels and then transferred to nitrocellulose membranes.

Nuclear and cytosolic extracts were prepared as previously described [19]; four times more of nuclear extract was used in order to detect ATX-3.

After incubation with the primary antibodies: anti-HSP16 (1∶5000, kindly provided by Dr. Christopher Link), anti-HSP40 (1∶2000, Stressgene), anti-alpha-tubulin (1∶100, DSHB), anti-histone H3 (1∶5000, Abcam), anti-ATX-3 (1∶200) the secondary antibodies were incubated at a 1∶10,000 dilution. Detection was done using ECL kit (Pierce). Band quantification was performed using ImageJ(http://rsbweb.nih.gov/ij/) as advised by the software manufacturers using alpha-tubulin as the loading control.

### Quantitative PCR

Total RNA from synchronized populations of *C. elegans* at various conditions was isolated using Trizol, following the manufacturer's instructions. Two micrograms of total RNA were DNAse treated (Amersham) and converted into cDNA using the iScript kit (Biorad). Q-PCR was performed using SyberGreen(Qiagen) and the Biorad q-PCR CFX96 apparatus. Actin was used as a housekeeping gene. We used relative quantification to determine the fold change difference between wild type and *atx-3* mutants, using the ΔΔCT method as described before [48].

### Mass spectrometry sample preparation

Proteomic lysis buffer (8 M urea, 2 M thiourea, 4% CHAPS, 100 mM DTT, 1 mM PMSF, protease inhibitors (Roche)) was added to the frozen pellets of young adult animals (72 h post hatching) in basal (20°C) or heat-shocked conditions (2 hours at 33°C with 30 minutes recovery at 20°C). Pellets were frozen and thawed several times and centrifuged. One hundred micrograms of total protein were precipitated using six volumes of cold acetone to remove interfering substances. Protein pellets wereressuspended in 40 µl of 500 mMtriethyl ammonium bicarbonate with 1% SDS, vortexed, and briefly sonicated until fully dissolved. The proteins were reduced using 2 µl of 50 mMtris-(2-carboxyethyl) phosphine for 1 hour at 60°C, and cysteines were blocked with 200 mM methyl methane thiosulfonate for 10 minutes at room temperature. Ten micrograms of sequencing grade modified trypsin (Promega) diluted in water was added to each sample and incubated overnight at 37°C. The iTRAQ reagents (Applied Biosystems) were reconstituted in 70% ethanol, transferred to the respective sample and allowed to incubate for one hour. Reagent 114 was added to N2, 115 to *atx-3(gk193)*, 116 to *atx-3(tm1689)*; and the labelled peptides were then combined and evaporated to dryness in a SpeedVac. Each sample was re-dissolved in 50 µl of 0,1% trifluoroacetic acid (TFA) and desalted using C18 empore disks.

### Isolelectric focusing

Desalted samples were applied on a 13 cm IPG strip, pH 3–10 (GE Healthcare), rehydrated for 12 hours and then focused in a IPGphorapparatus (GE Healthcare)using the following parameters: hold 500 V 1 h, linear gradient from 500–1000 V 15 minutes, hold at 1000 V 1 h, linear gradient from 1000 V–8000 V 30 minutes, hold 8000 2 hours. The strips were cut into 12 fractions and focused peptides were extracted from the gel using a TFA gradient. The sample were evaporated and desalted as mentioned above. Peptides were dissolved in 1% HCOOH just before LC-ESI-MS/MS analyses.

### LC-ESI-MS/MS analyses

Around 6–8 ug of peptides from each fraction were analyzed using a nanoflow LC coupled to a QSTAR Pulsar i ESI-hybrid Q-TOF tandem mass spectrometer (Applied Biosystems/MDS Sciex). Peptides were concentrated and desalted on a precolumn (0.3×5 mm C18 PepMap100, LC Packings), and eluted at 200 nl/min by increasing concentration of acetonitrile onto a self-packed C18 reverse phase column (75 µm×15 cm, Magic 5 µm 100 Å C18, MichromBioResources Inc.) A linear 60 min gradient from 98% solvent A (97.8% water, 2% acetonitrile, and 0.2% formic acid) to 35% solvent B (95% acetonitrile, 4.8% water, and 0.2% formic acid) was used.

Data from LC-MS/MS runs were converted to peak list files with the Analyst QS software (version 1.1). For QSTAR data, the acquisition parameters were: extract only +2 or +3 charged percursor ions, 300.000–1300.000 m/z range, with a minimum of 20 counts. Data from LC-MS/MS runs were converted to peak list files with the Analyst QS software (version 1.1).

### Data analysis and protein identification

MS/MS spectra generated was analyzed using Mascot software (version 2.1.02) using the Wormbasewormprep 178 (available in ftp://ftp.wormbase.org/pub/wormbase/genomes/elegans/sequences/protein/) as the reference database. Trypsin was set as the digestion enzyme with a maximum of one missed cleavage site. The mass accuracy for parent ions was set as 100 ppm, and 0.3 Da was used for the fragment ion mass tolerance. The default search settings used for quantitative analysis and protein identification were: trypsin cleavage with fixed MMTS modification of cysteine, iTRAQ labelling and variable methionine oxidation.

A 5% peptide false positive rate was chosen and only proteins identified by two or more peptides were selected.

### Statistical analysis

General comparisons were performed using ANOVA in the SPSS version 16 software. Direct comparisons of the brood size were performed using the T-test. For the plotting and analysis of the Kaplan-Meyer life span curves, GraphPad Prism version 5 was used. For PCR and immunoblotting analysis, a T-test was performed. A p-value of 0.05 was considered the cut off for statistical significance.

## Supporting Information

Table S1 **Lifespan results depicted in** **Figure 1** **.**
(PDF)

Table S2 **Proteomic results from LC-ESI-MS/MS analysis of two ataxin-3 knockout strains in a basal condition and after heat shock.**
(XLS)

Table S3 **Lifespan results depicted in** **Figure 7** **.**
(PDF)

Table S4 **Primers used in this study.**
(PDF)

Figure S1 **DAF-16 targets** ***sod-3*** **and** ***mtl-1*** **are activated differentially in** ***atx-3*** **mutants both at 20°C (A) and 25°C (B).** At 20°C, *sod-3* is up-regulated 90 minutes after heat shock while *mtl-1* is up-regulated 15 minutes after the stress. At 25°C, *sod-3* is up-regulated in all time points analyzed in *atx-3* mutants while *mtl-1* expression is increased starting at 15 min until 60 minutes after stimulus. *p<0.05.
(PDF)
