# Supplementary material for: Absence of Ataxin-3 Leads to Enhanced Stress Response in C. elegans
Source: PLoS One. 2011 Apr 19;6(4):e18512. doi: 10.1371/journal.pone.0018512 (PMC3079722; doi:10.1371/journal.pone.0018512)
Supplement: Table S1 — Lifespan results depicted in Figure 1 . (PDF) [file pone.0018512.s001.pdf]

Table S1

	Strain	n	Mean survival (hours)	p value
**1A**	N2	140	9	
	atx-3(gk193)	70	10	< 0.0001
	atx-3(tm1689)	70	10	< 0.0001
	daf-2(e1370)	39	nd*	
**1B**	N2	40	12.5	< 0.0001
	atx-3(gk193)	49	17	< 0.0001
**1C**	N2	70	9	
	atx-3(gk193)	70	10	< 0.0001
	N2 pre-HS	85	10	< 0.0001⁺
	atx-3(gk193) pre-HS	91	14	< 0.0001
**1D**	N2	70	9	
	atx-3(gk193)	71	10	< 0.0001
	N2 pre-HS	70	13	< 0.0001⁺
	atx-3(gk193) pre-HS	69	15	< 0.0001
**1E**	N2	40	12.5	
	atx-3(gk193)	49	17	< 0.0001
	N2 pre-HS	62	18	< 0.0001⁺
	atx-3(gk193) pre-HS	51	22	< 0.0001
**1F**	N2	40	12.5	
	atx-3(gk193)	49	17	< 0.0001
	N2 pre-HS	45	17	< 0.0001⁺
	atx-3(gk193) pre-HS	53	20	0.05

** not determined.*
⁺compared to N2 without pre-HS
p values for *atx-3(gk193)* pre-HS are obtained from comparison with N2 pre-HS
