# Supplementary material for: Absence of Ataxin-3 Leads to Enhanced Stress Response in C. elegans
Source: PLoS One. 2011 Apr 19;6(4):e18512. doi: 10.1371/journal.pone.0018512 (PMC3079722; doi:10.1371/journal.pone.0018512)
Supplement: Table S3 — Lifespan results depicted in Figure 7 . (PDF) [file pone.0018512.s003.pdf]

Table S3

	Strain	n	Mean survival (hours)	p value
7C	N2	22	12.5	
	atx-3(gk193)	40	17	< 0.0001
	daf-16(mu86)	28	13	0.5924
	gk193;mu86	47	13	0.4253[+]
	daf-2(e1370)	50	nd*	
7D	N2	49	17	
	atx-3(gk193)	37	19	0.0014
	daf-16(mu86)	51	16	0.7899
	gk193;mu86	22	17	0.2199[+]
	daf-2(e1370)	49	nd*	
7E	*daf-16* rescue + *L4440*	140	14	
	daf-16 rescue + RNAi(ATX-3)	202	16	<0.0001
7F	N2 + L4440	157	21	
	atx-3(gk193) + L4440	126	23	<0.0001
	N2 + RNAi(HSP-16.2)	151	19	0.04
	atx-3(gk193) + RNAi(HSP-16.2)	132	17	<0.0001[∀]
7G	N2 + L4440	152	14	
	atx-3(gk193) + L4440	87	16	<0.0001
	N2 + RNAi(C12C8.1)	137	14	0.0086
	atx-3(gk193) + RNAi(C12C8.1)	111	14	0.0035[&]
7H	N2 + L4440	91	13	
	atx-3(gk193) + L4440	42	15	0.0004
	N2 + RNAi(F44E5.5)	133	13	0.19
	atx-3(gk193) + RNAi(F44E5.5)	115	13	0.4021[#]

[+]compared to single mutant daf-16(mu86)
[∀]compared to N2 in RNAi(HSP-16.2)
[&]compared to N2 in RNAi(C12C8.1)
[#]compared to N2 in RNAi(F44E5.5)
