# Supplementary material for: Absence of Ataxin-3 Leads to Enhanced Stress Response in C. elegans
Source: PLoS One. 2011 Apr 19;6(4):e18512. doi: 10.1371/journal.pone.0018512 (PMC3079722; doi:10.1371/journal.pone.0018512)
Supplement: Table S4 — Primers used in this study. (PDF) [file pone.0018512.s004.pdf]

Table S4

Gene	Primer name	Sequence
HSP-4	HSP-4_F	TGACTCGTGCCAAGTTTGAG
	HSP-4_R	GCTCCTTGCCGTTGAAGTAG
HSP-16.1	HSP-16.1_F	GTCACTTTACCACTATTTCCGTCCAGCTCAACGTTC
	HSP-16.1_R	CAACGGGCGCTTGCTGAATTGGAATAGATCTTCC
HSP-16.2	HSP-16.2_F	TTGCCATCAATCTCAACGTC
	HSP-16.2_R	CTTTCTTTGGCGCTTCAATC
HSP-16.49	HSP-16.49_F	GCTCATGCTCCGTTCTCCATATTCTGATTCAAATGC
	HSP-16.49_R	GCAACAAAATTGATCGGAATAGAACGTGATGAG
F44E5.4	F44E5.4_F	CGTTTCGAAGAACTGTGTGCTGATCTATTCCGG
	F44E5.4_R	TTAATCAACTTCCTCAACAGTAGGTCCTTGTGG
C12C8.1	C12C8.1_F	GCTGATCTTTTCCGCAAGAC
	C12C8.1_R	CCAAAGGCTACTGCTTCGTC
ACT-1	ACT-1F	GTCGGTATGGGACAGAAGGA
	ACT-1R	GCTTCAGTGAGGAGGACTGG
HPRT-1	HPRT-1_F	TCCAACTCGTGCTGTCATTC
	HPRT-1_R	CGATTGGTTTGTTTCCGATT
ATX-3	ATX-3_F	GAAAATCCTGCGATGGTGGA
	ATX-3_R	ACTTTTGGCGGTACAACTGG
MTL-1	MTL-1_F	ATGGCTTGCAAGTGTGACTG
	MTL-1_R	CACATTTGTCTCCGCACTTG
SOD-3	SOD-3_F	CCAACCAGCGCTGAAATTCAATGG
	SOD-3_R	GGAACCGAAGTCGCGCTTAATAGT
