# Supplementary figures and images for: Absence of Ataxin-3 Leads to Enhanced Stress Response in C. elegans
Source: PLoS One. 2011 Apr 19;6(4):e18512. doi: 10.1371/journal.pone.0018512 (PMC3079722; doi:10.1371/journal.pone.0018512)

Figure S1

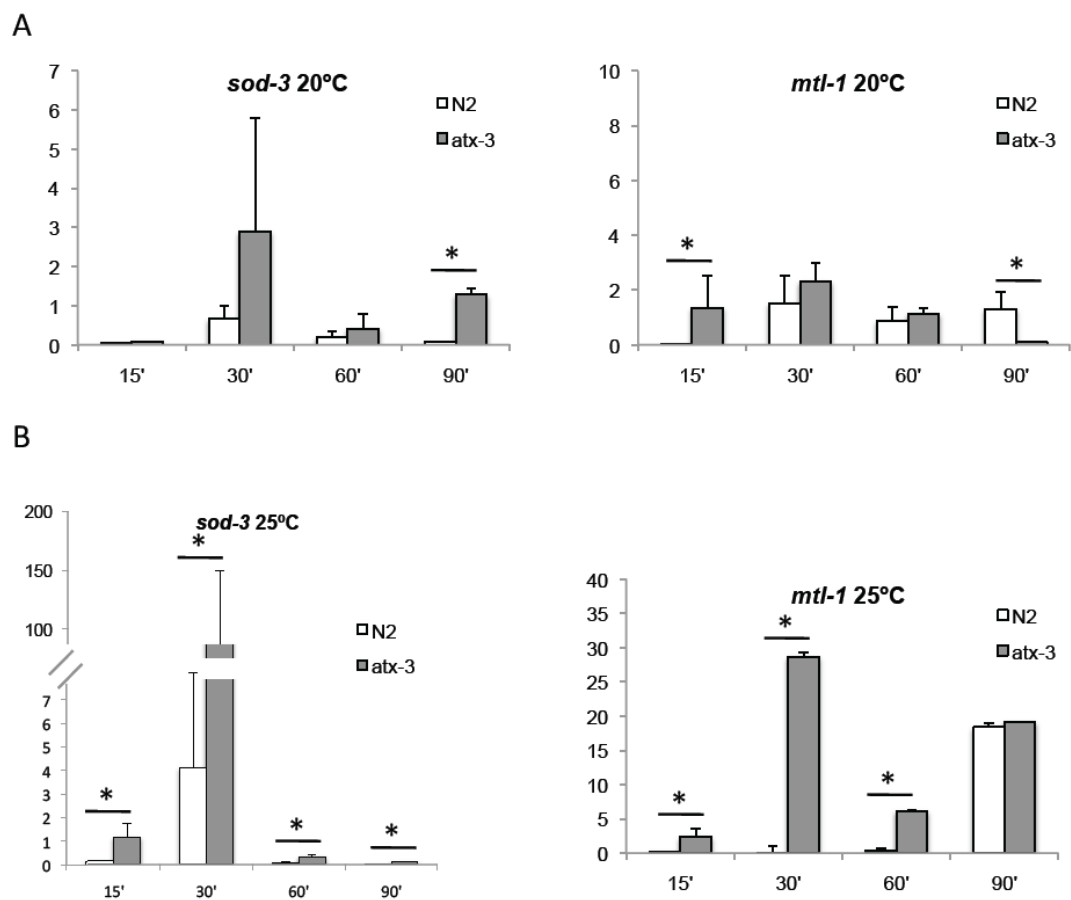

Supplement: Figure S1 — DAF-16 targets sod-3 and mtl-1 are activated differentially in atx-3 mutants both at 20°C (A) and 25°C (B). At 20°C, sod-3 is up-regulated 90 minutes after heat shock while mtl-1 is up-regulated 15 minutes after the stress. At 25°C, sod-3 is up-regulated in all time points analyzed in atx-3 mutants while mtl-1 expression is increased starting at 15 min until 60 minutes after stimulus. *p<0.05. (PDF) [file pone.0018512.s005.pdf]
